# Diversity of Bacterial Communities of Fitness Center Surfaces in a U.S. Metropolitan Area

**DOI:** 10.3390/ijerph111212544

**Published:** 2014-12-03

**Authors:** Nabanita Mukherjee, Scot E. Dowd, Andy Wise, Sapna Kedia, Varun Vohra, Pratik Banerjee

**Affiliations:** 1 Division of Epidemiology, Biostatistics, and Environmental Health, School of Public Health, The University of Memphis, 338 Robison Hall, 3825 Desoto Avenue, Memphis, TN 38152, USA; E-Mails: nmkhrje1@memphis.edu (N.M.); skedia1@yahoo.com (S.K.); vrnvhr22@gmail.com (V.V.); 2 Molecular Research LP (MR DNA), 503 Clovis Road, Shallowater, TX 79363, USA; E-Mail: sdowd@mrdnalab.com; 3 WMC TV Action News 5, NBC Memphis, 1960 Union Ave, Memphis, TN 38104, USA; E-Mail: awise@wmctv.com

**Keywords:** bacteria, microbiome, metagenomics, *Staphylococcus*, indoor environment, fitness center, gymnasium, hygiene, disinfection

## Abstract

Public fitness centers and exercise facilities have been implicated as possible sources for transmitting community-acquired bacterial infections. However, the overall diversity of the bacterial community residing on the surfaces in these indoor environments is still unknown. In this study, we investigated the overall bacterial ecology of selected fitness centers in a metropolitan area (Memphis, TN, USA) utilizing culture-independent pyrosequencing of the 16S rRNA genes. Samples were collected from the skin-contact surfaces (e.g., exercise instruments, floor mats, handrails, *etc.*) within fitness centers. Taxonomical composition revealed the abundance of *Firmicutes* phyla, followed by *Proteobacter* and *Actinobacteria*, with a total of 17 bacterial families and 25 bacterial genera. Most of these bacterial genera are of human and environmental origin (including, air, dust, soil, and water). Additionally, we found the presence of some pathogenic or potential pathogenic bacterial genera including *Salmonella*, *Staphylococcus*, *Klebsiella*, and *Micrococcus*. *Staphylococcus* was found to be the most prevalent genus. Presence of viable forms of these pathogens elevates risk of exposure of any susceptible individuals. Several factors (including personal hygiene, surface cleaning and disinfection schedules of the facilities) may be the reasons for the rich bacterial diversity found in this study. The current finding underscores the need to increase public awareness on the importance of personal hygiene and sanitation for public gym users.

## 1. Introduction

As we aspire to stay fit and healthy, many of us regularly visit fitness centers or “gyms”. In fact, the data from the International Health, Racquet & Sportsclub Association (IHRSA) indicates a surge in the number of people visiting fitness centers in the last five years [1]. In the year 2012, more than 58 million persons used health clubs in the U.S., in other words, one out of five (20%) Americans of ages 6 years and older are using health club facilities [2]. However, there is a lack of knowledge about the diversity of microbial communities at fitness centers. It is important to note that the overall microbial load and diversity of the environment are often implicated as a critical indicator of hygiene and cleanliness [3,4,5]. Several previous studies focusing on environmental hygiene and sanitation (e.g., in food production or health care settings) had found a direct relationship between microbial load in the surrounding environment and the risk of pathogen transmission [4,6,7]. Therefore, an understanding of overall bacterial population and diversity in gymnasiums and athletic facilities would obviously shed light on the risk of the pathogen propagation from these facilities. Most of the studies reported to date from gymnasiums, playgrounds, athletic facilities, or venues where individuals come in contact with others are focused on the transmission of staphylococci*,* in particular on methicillin-resistant *Staphylococcus aureus* (MRSA) [8,9,10,11,12,13,14]. It is also important to note that most of these studies relied on culture-based techniques [15]. Because a large number of microorganisms are difficult to culture [16], thus, the overall microbial diversity associated with fitness center environments remains largely unknown. The recent advancement of high-throughput sequencing techniques and related bioinformatics tools made it possible to envisage the microbial communities that inhabit humans and surroundings in great detail. By enabling identification of both “cultivable” and “non-cultivable” microbial populations, this culture independent method provides a vivid realization of the relationship among humans, microbes, and the environment. For example, microbial communities of several indoor environments, including hospitals [17], office buildings [18], kitchen [19], public restrooms [20], showers [21], have been documented. In most of the previous findings, human skin was found to be the main source of bacteria in each of the above-mentioned locations. However, other attributes such as soil and outdoor air (by air conditioning), dust from human shoes, *etc.* could also serve as potential sources of harboring and transmission of various microorganisms in indoor environments. Pathogenic microorganisms can survive on inanimate surfaces for prolonged periods of time as reported in several previous studies [22,23,24,25]. These pathogens can readily be transferred from surfaces to the human body through the touch of hands or other body parts. Carpets, yoga mat, clothes, equipment handles, *etc.* may serve as excellent living places for bacteria. The bacterial communities found on different surfaces are reported to be distinct [19]. Moreover, some studies delineated the role of environmental surfaces in the transmission of bacteria [26,27,28]. For instance, surfaces in public places namely, computers, telephones, telephone mouthpieces, headsets, desks, automated teller machines (ATM), cash machines, elevator buttons, are all reported as potential sources for transmitting infectious microorganisms [26,29,30,31,32]. 

Compared to other indoor environments, it is interesting to note that fitness centers offer a unique setting to explore microbial diversity. This can be attributed to the physical activities with high frequency of surface touch by individuals with different personal hygienic practices. Such factors are likely to have strong influences on the types of bacteria observed on fitness center surfaces. However, there is a lack of information on the microbial ecology of fitness centers in terms of the potential danger to the environment and public health. In the current study, we explored the overall bacterial ecology of selected fitness centers in a metropolitan area (Memphis, TN, USA) utilizing culture-independent pyrosequencing of the 16S rRNA genes. Our goal was to assess and comprehensively understand the microbial diversity associated with fitness center surfaces; and to determine if different surfaces of fitness centers (e.g., exercise instruments, floor mats, handrails, *etc.*) serve as potential reservoirs for different bacterial communities. 

## 2. Experimental Section 

### 2.1. Sample Collection

For this study, surface swab samples were collected from four membership-based fitness centers around the Memphis metropolitan area in Tennessee. Two out of the four fitness centers are open 24 h/7-days a week. Samples were collected by trained volunteers from the skin-contact surfaces on exercise equipment (nautilus machine, treadmill, stationary bike, power strider, elliptical machine, and leg press), dumbbell, toilet handles, and handrails on stairs of the fitness centers during October 2013. The samples were obtained from certain places that had not been sanitized before sample collection. Cotton-tipped swabs (Sanicult^TM^, Thermo Remel/ Starplex Scientific Inc., Etobicoke, ON, Canada) were used to wipe the surface (approximately 10 cm^2^) by rotating over 4–5 times (~10 s). While swabbing on equipment surfaces with different shapes, appropriate care was taken to cover approximately the same surface area. After swabbing, the swab sticks were immediately placed back into the tube containing sterile diluent solution and the samples were transported in a refrigerated container to the laboratory within four hours for analysis.

### 2.2. DNA Extraction and Pyrosequencing

Genomic DNA was extracted from the swab samples using the DNA extraction kit (MO BIO Laboratories, Carlsbad, CA, USA), following the manufacturer’s protocol for isolation of DNA from microbial cultures. The extracted genomic DNA samples were quantified spectrophotometrically using a NanoDrop spectrophotometer (Thermo Scientific, Wilmington, DE, USA). The DNA samples were pooled based on the types of equipment and surfaces and subjected for pyrosequencing. The details of pooling swab samples is described in Table A1 (Appendix Material).

In this study, we performed bTEFAP^®^ (MR DNA www.mrdnalab.com) which was originally described by Dowd *et al.* [33,34] and that has been utilized to describe a wide range of environmental- and health-related microbiomes [33,34,35]. To evaluate the microbial ecology of samples, 16S universal Eubacterial primers 27Fmod (AGRGTTTGATCMTGGCTCAG) and 519Rmod (GTNTTACNGCGG CKGCTG) were utilized on the Illumina MiSeq v3 2 × 300 bp sequencing platform (Illumina, San Diego, CA, USA). A single-step 30 cycle PCR using HotStarTaq Plus Master Mix Kit (Qiagen, Valencia, CA, USA) were used under the following conditions: 94 °C for 3 min, followed by 28 cycles of 94 °C for 30 s; 53 °C for 40 s and 72 °C for 1 min; after which a final elongation step at 72 °C for 5 min was performed. After the completion of PCR, all amplicon products from different samples were mixed in equal concentrations and purified using Agencourt Ampure beads (Agencourt Bioscience Corporation, Beverly, MA, USA).

### 2.3. Computational and Statistical Analyses

The Q25 sequence data derived from the sequencing process was processed using a proprietary analysis pipeline (www.mrdnalab.com, MR DNA, Shallowater, TX, USA). Sequences were depleted of barcodes and primers with short sequences (<200 bp), sequences with ambiguous base calls, and sequences with homopolymer runs exceeding 6 bp were removed. Sequences are then denoised and chimeras removed. Operational Taxonomic Units (OTUs) were defined after the removal of singleton sequences, clustering at 3% divergence (97% similarity). [33,34,36,37,38]. OTUs were then taxonomically classified using BLASTn against a curated GreenGenes/RDP/NCBI derived database [39] and compiled into each taxonomic level into both “counts” and “percentage” files. Counts files contain the actual number of sequences while the percent files contain the relative (proportion) percentage of sequences within each sample that map to the designated taxonomic classification. For example, if there are 1000 sequences and 100 of the sequences are classified as *Staphylococcus* then it has been represented as *Staphylococcus* being 10% of the total population. Statistical analysis in this study was performed using a variety of computer packages including XLstat, NCSS 2007, “R” and NCSS 2010. Alpha and beta diversity analysis was conducted as described previously [33,34,36,37,38] using QIIME (Quantitative Insights Into Microbial Ecology). Significance reported for any analysis is defined as *p* < 0.05.

## 3. Results 

### 3.1. Diversity and Relative Abundance of Bacterial Genera in Surface Swab Sample of Fitness Center

DNA extraction from all surface swab samples contained measurable quantities of microbial DNA (48–152 ng/µL); negative controls (no cells) had no quantifiable DNA. After stringent quality sequence curation, a total of 122,454 sequences were generated by the bTEFAP^®^ of DNAs from thirty two surface samples (swabs). A total of 111,663 sequences were utilized for the analyses of the eleven sample groups (which were based on the equipment types sampled, including the pooled samples from week 1 and 2). The details of pooling samples and clustering into groups have been described in Table A1. 

**Figure 1:**
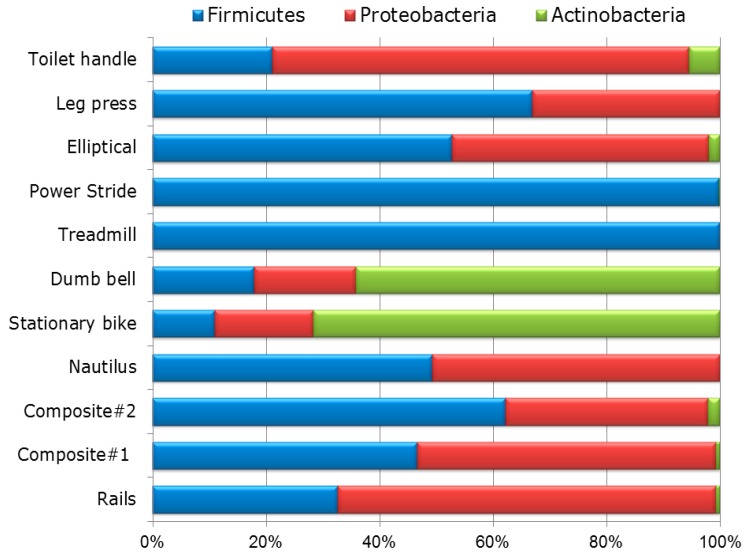
Relative abundance of bacterial diversity of different surfaces of gym equipment at phylum level as determined by bTEFAP^®^. Bacterial diversity varied between equipment or surface types. *Firmicutes* are found to the predominant phyla, followed by *Proteobacteria* and *Actinobacteria* phyla The least bacterial diversity at phylum level was found in surfaces of power striders and treadmills which consisted mostly of *Firmicutes* (>99%).

**Figure 2:**
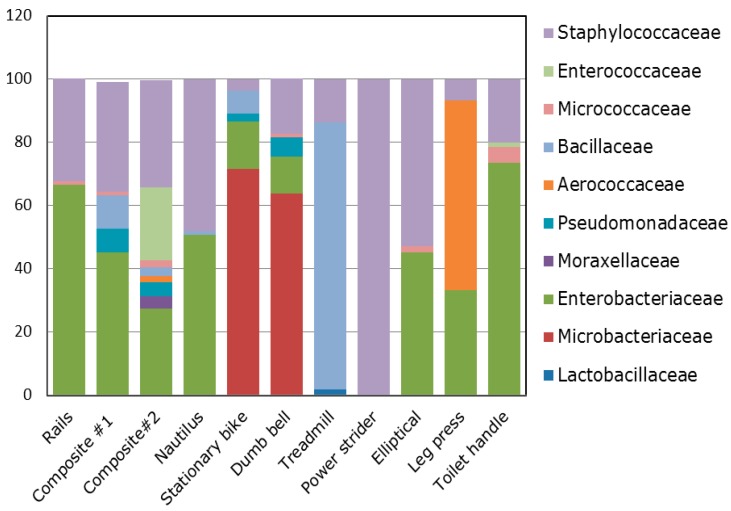
Relative abundance of bacterial families of different surfaces of gym equipment as determined by bTEFAP^®^. Multi-colored stack bar graphs represent the relative abundance of bacterial family in each sample.

Taxonomical composition indicated that the *Firmicutes* phyla was most common, followed by *Proteobacteria* and *Actinobacteria* phyla (Figure 1) with a total of 25 identified genera and 63 species across all surface swab samples. Within these dominant phyla, bacterial family associated with mostly human normal flora and environmental bacteria such as *Staphylococcaceae*, *Enterobacteriaceae*, *Microbacteriaceae*, *Bacillaceae*, *Aerococcaceae*, and *Pseudomonadaceae* have been found abundantly in all surface swab samples (Figure 2). 

**Figure 3:**
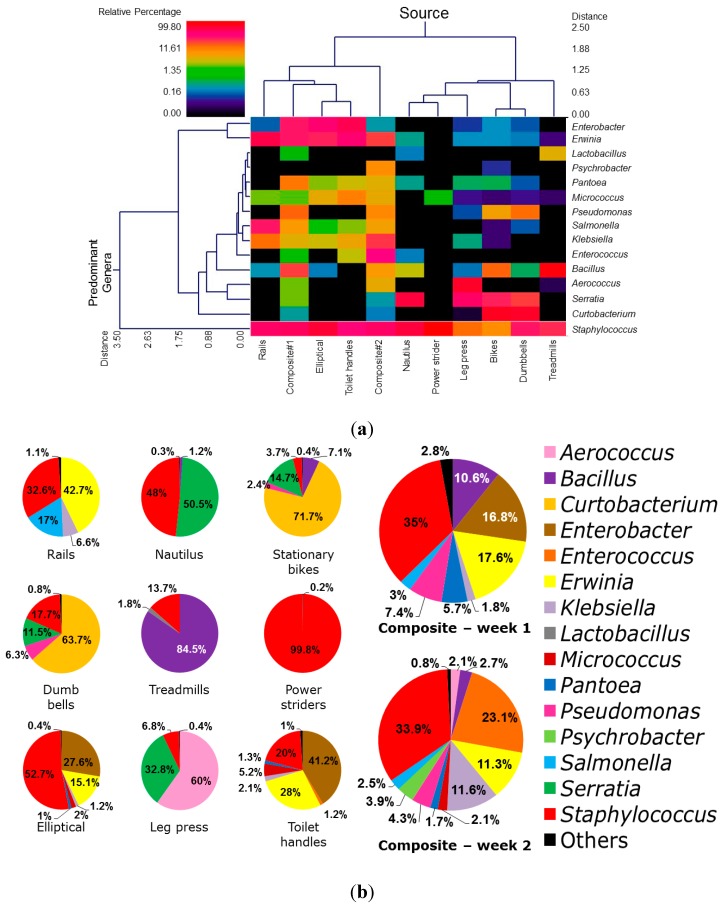
(**a**) Dual hierarchical clustering dendogram of the bacterial genera profiles for fitness center equipment and items. Samples with more similar microbial populations are clustered closer together. The top ~15 most abundant genera (average across all samples) are used for clustering. The heatmap represents the relative percentages of each bacterial genus. The predominant genera are represented along the right Y-axis, while each sample (surface swabs from equipment and items) is clustered on the X-axis. The legend for the heatmap is provided in the upper left corner. (**b**) Relative abundance of bacterial diversity at the genus level in the surface swabs of gym equipment. Bacterial genus abundance less than 1% were grouped as “Others”.

The relative abundance of bacterial diversity in genus level is presented in Figure 3. A high abundance of *Staphylococcus* spp. was observed in most of the samples. Some other bacterial species such as, *Bacillus*, *Serratia*, *Aerococcus*, *Erwinia,* and *Enterobacter* spp. were observed predominantly on treadmills, nautilus machines, leg press, rails (handrail on stairs), elliptical, and toilet handles, respectively. Some previously unreported bacterial genera associated with the surfaces of equipment in the fitness center have been identified in this study, namely *Klebsiella*, *Lactobacillus*, *Salmonella*, *Curtobacterium*, *Pantoea*, *Psychrobacter*, *Serratia*, *Bacillus*, *Pseudomonas*, *Micrococcus*, *Enterococcus*, *Erwinia*, and *Aerococcus.* The most prevalent bacterial species identified across all the samples observed were staphylococci*,* including *S. aureus*, *S. epidermidis,* and *S. saprophyticus*. In this study, *Staphylococcus* were found predominantly in power striders (99.8%), followed by elliptical machines (52.7%), and nautilus machines (48%), rails (32.6%), toilet handles (20%), dumb bells (17.7%), treadmills (13.6%), leg presses (6.8%), stationary bikes (3.7%), composite samples of week 1 (34.7%) and composite samples of week 2 (33.9%). Interestingly, *S. aureus* and *S. epidermidis* were found to be present on all the surfaces tested. 

A total of 25 bacterial genera were detected in all swabs sampled in this study. The predominant bacterial genera in the surface swab samples analyzed based on the relative abundance cutoff of 1.0% are shown in Figure 3b. The major bacterial genera include *Pseudomonas*, *Pantoea*, *Micrococcus*, *Staphylococcus*, *Enterobacter*, *Klebsiella*, and *Bacillus* (Figure 3). In addition, our results indicate that other prevalent bacterial genus such as *Erwinia* spp. were found on rails (42.7%) and in the composite samples from week 1 (17.6%). *Enterococcus* was prevalently found in the composite samples from week 2 (23.1%), whereas *Serratia* spp. was abundant on nautilus machines (50.5%). *Curtobacterium* spp. were observed on stationary bikes and dumb bells with an abundance of 71.7% and 63.7%, respectively. The air-borne bacteria *Aerococcus* spp. was prevalent on leg press machines (60%). Among the swab samples from treadmills, *Bacillus* spp. was the most abundant (84.5%) genus, whereas *Enterobacter* spp. was observed in elliptical machines and toilet handles with the relative abundance of 27.6% and 41.2%, respectively.

**Figure 4:**
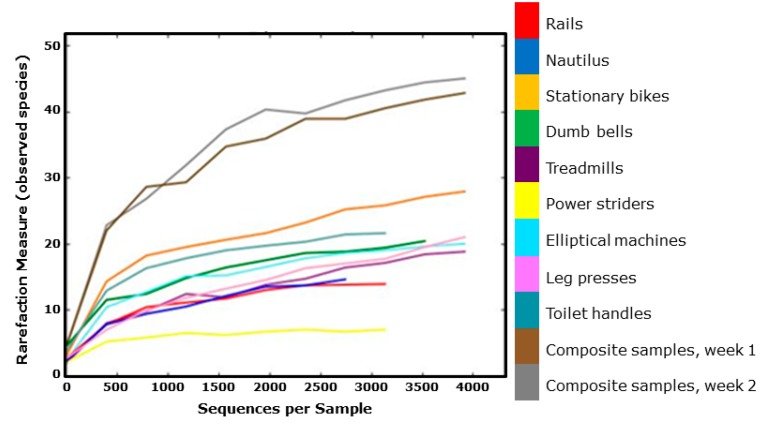
Observed taxonomic units analysis (Rarefaction Curve) of bacterial species diversity of surface swab samples in fitness centers. This curves show that as might be expected the composite samples from weeks 1 and 2 have the highest observed species while power Striders had the lowest observed species values.

Samples were rarefied to 2000 sequences for alpha and beta diversity and bootstrapping analyses. The indices of bacterial diversity were estimated using a Rarefaction Curve (Figure 4) based on OTUs. Rarefaction Curve modeling indicated 97% similarity of OTUs at the 3% divergence was attained for each sample [33,34,36,37,38] suggesting adequate depth of coverage. By rarefaction analysis estimates (Figure 4), the trend for species richness in different equipment surfaces was found to be (high to low): stationary bikes > toilet handles > dumb bells > elliptical machines > leg presses > treadmills > nautilus machines > stair rails > power striders. As expected, the composite samples showed the highest bacterial species diversity since they were pooled DNA of all samples for a specific sampling week. 

**Figure 5:**
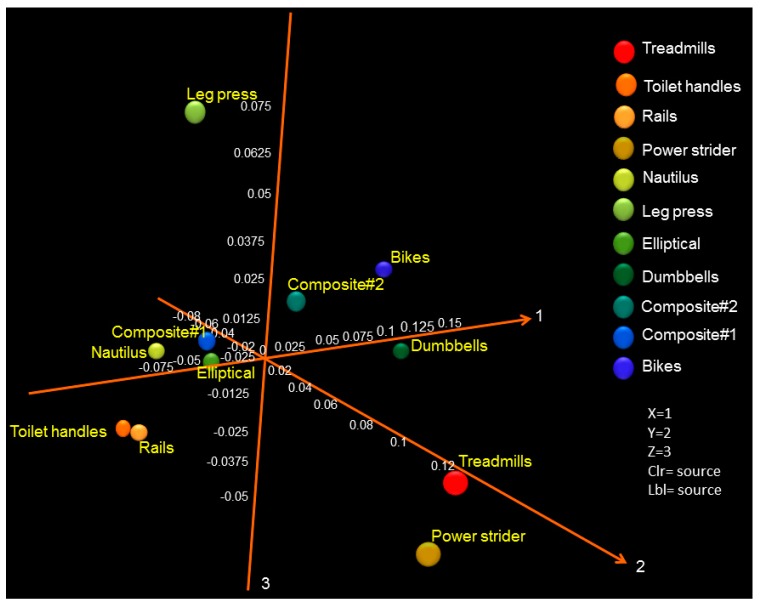
PCoA analysis of the microbiome of each equipment and items swabbed based upon UniFrac method. Different colored symbols are indicative of the various gym equipment and items. Symbols that are closer together represent similar surface bacterial communities.

Using weighted Principal Coordinates Analysis (PCoA) of the microbiome of each sample based upon UniFrac method, we see that the samples most distant from the central cluster are leg press, treadmills, and power striders (Figure 5). Toilet handles and rails to the stairs cluster together, bikes and dumbbells cluster near to each other and the nautilus and elliptical machines group together.

### 3.2. Probable Source of Bacteria in Surface Swab Sample of Fitness Center

**Figure 6:**
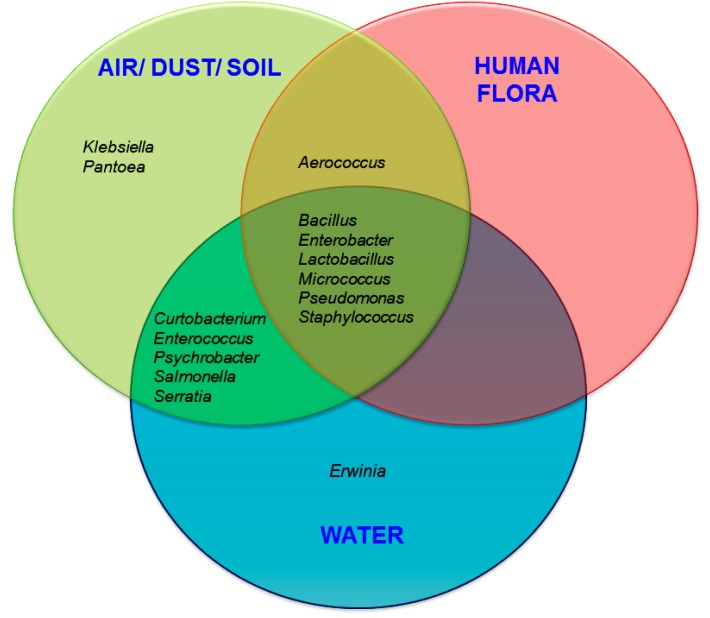
Results showing the potential contributions of different sources to the fitness center surface associated bacterial communities.

The tentative source/habitat of the bacteria has been shown in Figure 6. The probable environmental sources/habitats of bacteria were classified based on previous reports/literature [20,40,41]. It is evident from our results that bacterial genera commonly associated with air/soil/dust-borne transmissions contribute to a relatively high proportion of the bacterial community in the fitness centers. They include *Aerococcus*, *Bacillus*, *Curtobacterium*, *Paenibacillus*, *Pantoea*, *Pseudomonas*, *Psychrobacter*, and *Serratia*.

Human associated bacteria (human flora) were found in this study are Aerococcus, Bacillus, Enterobacter, Lactobacillus, Micrococcus, Pseudomonas, and Staphylococcus [42]. Bacterial genera such as Aerococcus, Bacillus, Enterobacter, Lactobacillus, Micrococcus, Pseudomonas, and Staphylococcus share their habitat in three clusters as depicted in Figure 6. Additionally, many of these genera, namely, Aerococcus, Bacillus, Curtobacterium, Enterobacter, Enterococcus, Lactobacillus, Micrococcus, Pseudomonas, Psychrobacter, Salmonella, Serratia, and Staphylococcus have been reported previously from both air/dust/soil and water [20,40,41]. 

Notably, the presence of some pathogenic (or potentially pathogenic) bacterial genera were observed in relatively low abundance, including *Bacillus* in composite samples from week 2 (2.7%), nautilus machine (1.2%), stationary bike (7.1%); *Enterococcus* (1.2%) on the toilet handle; *Klebsiella* on rails (6.6%), composite samples from week 1 (1.8%), elliptical machines (1.2%), toilet handle (2.1%); *Pantoea* in composite samples from week 1 (5.7%), composite samples from week 2 (1.7%), elliptical machines (1%), toilet handle (1.3%); *Pseudomonas* in composite samples from week 1 (7.4%), composite samples from week 2 (4.3%), stationary bike (2.4%), dumb bell (6.3%). Moreover, it is interesting to note that pathogenic *Salmonella* spp. were found on rails (17%), composite samples from week 1 (2.6%), and in composite samples from week 2 (2.5%). 

## 4. Discussion and Conclusions 

This study explored the bacterial loads and diversity associated with different equipment surfaces in fitness centers using culture independent sequencing based methods. Taxonomical composition revealed that the predominant phyla (in terms of percentages and read) were *Firmicutes*, *Proteobacteria* and *Actinobacteria*. Prevalence of these phyla have also been reported previously from various indoor environments utilizing both culture-dependent and independent techniques [43,44,45]. Within these dominant phyla, the bacterial families with the highest relative abundances across all the samples were *Bacillaceae*, *Staphylococcaceae*, *Enterobacteriaceae*, *Aerococcaceae*, and *Microbacteriaceae*. In general, the most common bacterial genus observed in this study was found to be *Staphylococcus*. The presence of *Staphylococcus* has been frequently reported from gymnasium, playground, beach, college, daycare centers, athletic facilities, where person to person contact may occur [8,9,10,11,12,13,14]. In the present study, we identified the presence of several *Staphylococcus* spp. (tentatively, *S. aureus*, *S. cohnii*, *S. epidermidis*, *S. haemolyticus*, *S. hominis, S. pasteuri*, and *S. saprophyticus*) in all surface swab samples. Among them, *S. saprophyticus* is the most predominant bacterial species, followed by *S. epidermidis*, and *S. aureus*. Pathogenic *S. saprophyticus*, commonly present in the human urogenital and gastrointestinal tract, in food products such as cheese, meat, and vegetables, and in the environment, has been associated with urinary tract infections (UTI), particularly in young women [46,47,48,49,50,51]. Human behaviors such as outdoor swimming, sexual intercourse, and work in meat production may be associated with UTI caused by *S. saprophyticus* [52]. *S. epidermidis*, a human skin flora [53] also been considered as an opportunistic pathogen, can cause several health problems, including bacteremia, surgical wound, dialysis-associated and prosthetic-joint infections [54,55]. Production of biofilms, implicated as a major virulence factor, protects *S. epidermidis* from antibiotics and host immune defenses [55,56,57]. *S. epidermidis* exhibits resistance to multiple antibiotics, including methicillin and many additional antibiotics [58]. One of the most infamous antibiotic resistant strains among staphylococci, MRSA, has been known to transmit from people to people by skin contact, fomite to people contact, or through touching of contaminated surfaces [59,60]. The transmission of *S. aureus* has been reported from the public places such as gymnasiums, playgrounds, beaches, schools, daycare centers, and athletic facilities [8,9,10,11,12,13,14]. Moreover, MRSA was also identified in indoor environments such as kitchen and bathroom surfaces [61]. Interestingly, *S. aureus* can survive on inanimate surfaces for a long time [23,24,25]. The human infection related to community-associated MRSA (CA-MRSA) is distributed widely throughout the world [62]. Moreover, *S. aureus* is implicated to skin and soft-tissue infections (SSTI). In USA, the prevalence of SSTI is increasing. In 2005, there were more than 14 million outpatient clinic visits for SSTI (~50 visits/1,000 in the US) compared with only 8.6 million visits (~32 visits/1000) in 1997 [63]. Due to this substantial burden, MRSA is a top priority for the Institute of Medicine’s Comparative Effectiveness Research Program [64]. The presence of *S. aureus* in this study is an obvious public health concern. Future study is needed to evaluate the prevalence of antibiotic resistance of *S. aureus* isolates obtained in the present study. However, the high prevalence of *Staphylococcus* spp. in these samples is not surprising as most of these species are part of human normal flora. 

Most of the bacteria found in this study belong to environments such as, soil, dust, air and water and human flora. The prevalence of human flora and environmental bacteria on the swab samples is not surprising as most gym equipment surfaces frequently come into contact with human skin. In addition, many other human-associated bacteria, including several lineages associated with the gut, mouth, and urine, (e.g., *Klebsiella pneumoniae*, *Enterobacter faecalis*, *Staphylococcus saprophyticus*, *Staphylococcus aureus, Staphylococcus epidermidis*, *etc.*) were observed on the surfaces of toilet handles, which is also not surprising. 

Some pathogenic or potentially pathogenic bacteria such as tentatively *Salmonella enterica*, *Klebsiella pneumoniae*, *Enterococcus faecalis*, *Baciluus cereus*, *Pantoea agglomerans* have beendetected in swab samples. The presence of food-borne pathogenic bacteria *Salmonella enterica* (belonging to the bacterial family *Enterobacteriaceae*), associated with cattle and poultry [65], have been observed on stair rails, and in the composite samples from week 1 and 2. The probable reasons of the presence of *Salmonella enterica* in our study may be attributed to gym users who are either exposed to or come in contact with livestock or work in a veterinary clinic or having prior exposures to the infection source. Another pathogenic bacteria, *Klebsiella pneumoniae* (belonging to the bacterial family *Enterobacteriaceae*), associated with urinary tract infections [66] and bacteremic liver abscess [67], have been identified in our study. The presence of these bacteria may also be a public health concern.

Previous studies established that *Staphylococcus* and *Micrococcus* spp. are the most common bacteria found in indoor air environments, although *Aeromonas* spp. and some other bacteria belonging to the family *Pseudomonadaceae* are often present in indoor air environments [68,69]. Human normal flora *M. luteus* and *S. saprophyticus* have also been reported from the indoor environment [70]. Some dust-borne potential/opportunistic pathogens which are previously reported from indoor environments [71] were also identified in the current study including *Pseudomonas*, *Pantoea*, *Micrococcus*, *Staphylococcus*, *Enterobacter*, *Klebsiella*, and *Bacillus*. In addition, the air-borne bacterium *Aerococcus viridans*, which has previously been identified from the air of occupied rooms [45] was identified in high abundance on leg press equipment in this study. 

The survival of microorganisms and the microbial diversity in indoor environments depends on indoor ventilation design, air circulation, and relative humidity (RH) [72]. The bacteria capable of transmission through aerosols such as, *Pseudomonas*, *Enterobacter*, *Erwinia*, and *Klebsiella* species can survive in high RH and in low temperature [73,74]. *Serratia marcescens*, identified in high abundance from nautilus machines, stationary bikes, dumb bells, leg press, treadmills, and composite samples from weeks 1 and 2 in this study, cannot survive in high RH (70%–90% RH) environments [75]. While another pathogenic bacteria, *Klebsiella pneumoniae*, identified from rails and stationary bikes in this study, has been known to survive at 60% RH [76]. The probable reason of the presence of these RH sensitive bacteria may be due to the transport from a different place to fitness centers by several routes of entry, and these may reflect both climatic and personal hygiene influences.

Although the bacteria communities identified in this study can be transferred by surface touch, it is difficult to estimate the risk of acquiring the disease through surface touch as there are no reports of any associated diseases. Such reporting is rare, unless associated with a large epidemic outbreak. Here, the identification of bacterial communities was performed using DNA-based pyrosequencing methods, which reveal bacterial presence regardless of their viability status. These methodologies report non-viable or non-culturable cells along with culturable cells. Future study employing RNA-based methods, such as RNAsec, is required to confirm the presence of viable bacterial communities in the fitness centers. Other functional information such as presence of pathogenic determinants can be obtained by conducting real-time PCR-based assays or bioassays. Nevertheless, the current study provides a comprehensive assessment on the diversity in bacterial communities in the fitness center along with the knowledge of the potential presence of pathogenic organisms. Overall, our study represents the microbiome of selected fitness centers from metropolitan Memphis area (representing approximately 1.2 million populations) in Tennessee, USA, which can be deemed as a representative model of a large metropolitan setting. As revealed by our study, a high degree of microbial diversity originating from inanimate surfaces of fitness centers may be alarmingly implicated to poor personnel hygiene of facility users as well as to the inadequate cleanliness of the facilities. To conclude, it is critical to underscore the need of proper hygienic practices in fitness centers and gyms for minimizing the spread of disease-causing organisms.

## Appendix

**Table A1:** The detail of pooling swab samples.

Group	Description	Number of Samples (n) *
1	Stair rail (Rails)	2
2	Nautilus machine	4
3	Stationary bike	4
4	Dumb bell	4
5	Treadmill	4
6	Power Stride	4
7	Elliptical	4
8	Leg press	4
9	Toilet handle	2
10	Composite sample, week 1 ******	
11	Composite sample, week 2 ******	
Total		32

***** Samples were collected from four fitness centers with repeat sampling for two consecutive weeks. For bTEFAP^®^, DNA samples were pooled based on the types of equipment and surfaces resulting in nine (9) equipment/item groups. ****** Two additional groups of samples (Composite samples) were created by pooling all samples from week 1 and week 2 (total 11 sample groups).
